# Rapid guide to the management of cardiac patients during the COVID-19 pandemic in Egypt: “a position statement of the Egyptian Society of Cardiology”

**DOI:** 10.1186/s43044-020-00061-5

**Published:** 2020-05-27

**Authors:** Sameh Shaheen, Omar Awwad, Khalid Shokry, Magdy Abdel-Hamid, Adel El-Etriby, Hosam Hasan-Ali, Islam Shawky, Ahmad Magdy, Gamila Nasr, Hamza Kabil, Amr Elhadidy, Mohamad Zaki, Ahmad Hegab

**Affiliations:** 1 Faculty Of Medicine, Ain Shams University, Cairo, Egypt; 2 Faculty Of Medicine, Military Academy, Cairo, Egypt; 3 Faculty Of Medicine, Cairo University, Cairo, Egypt; 4 Faculty Of Medicine, Assuit University, Assuit, Egypt; 5 Faculty Of Medicine, Azhar University, Cairo, Egypt; 6 National Heart Institute, Giza, Egypt; 7 Faculty Of Medicine, Suez Canal University, Ismailia, Egypt; 8 Faculty Of Medicine, Banha University, Banha, Egypt

**Keywords:** COVID-19, Pandemic, Egypt, Cardiovascular disease

## Abstract

COVID-19 pandemic poses an enormous challenge to healthcare system in Egypt. This document is a position statement from the Egyptian Society of Cardiology. It aims to provide information to cardiovascular healthcare providers in Egypt to guarantee delivery of quality *patient care* and ensure adequate levels of *protection* against infection during the COVID-19 pandemic. Older patients and those with cardiovascular disease are at higher risk of mortality. The current situation requires unusual allocation of resources which may negatively impact the care of patients with cardiovascular disease. Cardiologists should be prepared in the COVID-19 pandemic. The challenge is in providing the best quality of care despite limited resources while keeping all medical staff as safe as possible. Consider deferring elective procedures whenever possible. All medical staff should undergo rigorous training on infection control and the use of high-quality personal protection equipment. Cardiologists should promote telemedicine in the outpatient setting, prioritize outpatient contacts, and avoid nosocomial dissemination of the virus to patients and healthcare providers. A much conservative approach for emergent cardiac patients is recommended, and invasive interventions are reserved for high risk hemodynamically unstable patients. During the pandemic, the most important principles of treatment should be controlling the spread of infection as the first priority, prompt assessment of patient risk, recommending conservative medical therapy rather than invasive interventions, and strict infection control measures to limit infection spread within the hospital and to healthcare workers.

## Background

COVID-19 pandemic poses an enormous challenge to healthcare system in Egypt. Older patients and those with cardiovascular disease are at higher risk of mortality. The current situation requires unusual allocation of resources which may negatively impact the care of patients with cardiovascular disease [1–3]. This document is a position statement from the Egyptian Society of Cardiology. It aims to provide information to cardiovascular healthcare providers in Egypt to guarantee the delivery of quality *patient care* and ensure adequate levels of *protection* against infection during the COVID-19 pandemic.

## Main text

### COVID-19 pandemic and cardiovascular disease patients

- During the COVID-19 pandemic, cardiologists might deal with these scenarios:

A. Cardiac patient with symptoms suspicious of COVID-19.
B. Undiagnosed patient with symptoms suspicious of COVID-19.
C. COVID-19 patient with cardiac complication.

- COVID-19 patients with cardiac problems are at higher risk of morbidity and mortality. They might have the following cardiac complications: exacerbation of previous cardiac problem, acute heart failure, acute myocarditis, acute coronary syndrome, acute stent thrombosis, venous thrombo-embolism, and various forms of arrhythmias. Some patients may present with ECG findings of ACS but with non-significant lesions. Other patients may present with severe cardiomyopathy and normal coronaries (Takotsubo-like syndrome). Some patients might suffer from side effects of COVID-19 treatment like hydroxychloroquine-azithromycin combination which might cause fatal prolonged QT interval [4–8].
- These patients should be investigated as follows: CBC, ESR, CRP, D dimers, cardiac troponin, ECG (to assess ischemia, arrhythmia, and QT interval), CXR (to assess signs of cardiomegaly or pneumonia), echocardiography (to assess LV diastolic dysfunction, LVEF, valvular lesions, and pericardial effusion), coronary angiography if indicated, PCR to nasopharyngeal swabs and CT chest. (Table 1 and Table 2)
- Most cardiac drugs, such as antiplatelets, statins, and RAS blockers can be safely continued after the diagnosis of COVID-19 [4–8].

**Table 1:** Recommended investigations for COVID-19

CBC	Leukopenia, leukocytosis, and lymphopenia
CRP	Elevated
Lactate dehydrogenase and ferritin	Elevated
Liver enzymes: SGOT, SGPT	Elevated
Urea, creat	Elevated
PT, PTT, INR	Elevated
D dimer	Elevated
C&S	May be bacterial infection
Cardiac enzymes	ELEVATED
ABG	HYPOXIA
CXR	may reveal pulmonary infiltrates
Nasoharyngeal swab for PCR. The test is a real-time reverse transcription-polymerase chain reaction (rRT-PCR) assay that can be used to diagnose the virus in respiratory and serum samples from clinical specimens.	The highest rates of positive results included BAL fluid (14/15; 93%), sputum (75/104; 72%), nasal swabs (5/8; 63%), brush biopsy (6/13; 46%), pharyngeal swabs (126/398; 32%), feces (44/153; 29%), blood (3/307; 1%), and urine (0/72; 0%). Nasal swabs were found to contain the most virus.
CT CHEST	Peripheral distribution (80%) Ground-glass opacity (91%) Fine reticular opacity (56%) Vascular thickening (59%) Central and peripheral distribution (14%) Pleural effusion (4.1%) Lymphadenopathy (2.7%)
ECG	Ischemia, arrhythmias, conduction delays
ECHO	Diastolic dysfunction LV systolic dysfunction Myocarditis, endocarditis, pericarditis

**Table 2:** Recommended investigations with definitive sensitivity and specificity for disease diagnosis or assessment

Acute aortic syndrome	CT angiography (CTA)
Acute pulmonary embolism	CT angiography (CTA), D-dimer testing and deep vein ultrasound in the lower extremity
Acute coronary syndrome	Ordinary ECG and standard biomarkers for cardiac injury
Cardiac mechanical complications	Bedside echocardiography
All patients should undergo lung CT examination	
Chest X-ray is not recommended because of a high rate of false-negative diagnosis.	

Modified from CSC expert consensus [11]

### Principle recommendations during COVID-19 pandemic

- During the pandemic, hospitals should be divided into two main categories:

A. *COVID-19 designated hospitals*—for patients infected with the virus. These hospitals will need all specialties including cardiologists.
B. *Non-COVID-19 designated hospitals*—for non-infected patients with other diseases. Yet, due to the long incubation period of the virus and the presence of asymptomatic infection, the potential infection risk of medical staff in non-designated hospitals exists. Moreover, some COVID-19 patients may present with what looks like cardiac complaints and this puts cardiologists at risk of getting the infection [9–11].

- Consult with the infection control unit in your hospital to establish a management system that minimizes nosocomial infections. Separate workers into groups so that possible quarantines can be applied to groups within each unit rather than the unit as a whole. All medical teams should also discuss and implement backup policies and schedules, in case that one staff member becomes ill or is quarantined and cannot participate in clinical coverage for a period of time.
- For invasive procedures, a single cath lab should be designated for the care of COVID-19 patients. Consider deferring elective procedures whenever possible. Deferral minimizes the risk of exposure to COVID-19 for patients and staff, and maximizes the availability of inpatient beds in anticipation of a surge in hospitalization required for COVID-19 infected patients.
- High-quality personal protection equipment (PPE) should be provided to all the staff dealing with these patients. All medical staff should undergo rigorous nosocomial infection training before they start work. The steps for donning and doffing PPE are critically important.
- It may be desirable to minimize medical staff older than 65 years and those with chronic diseases from being directly exposed to presumed or confirmed COVID-19 cases. The government should provide special free accommodation facilities for medical staff participating in the management of COVID-19 during the pandemic to minimize the dissemination of infection to their family members if they return home.
- Inpatients can only be accompanied by at most one family member who must complete the COVID-19 investigation and should wear face masks. Daily monitor body temperature and screen for COVID-19 related symptoms. No other visits during hospitalization [9–11].

### Outpatient management of cardiac patients

- Because some symptoms of COVID-19 look like those due to cardiovascular disease exposure risk exists in outpatient settings. Telemedicine via telephone or video calls can be used to triage patients as regards the need for face to face consultation.
- All medical staff should put on proper PPE (including gloves, protection suits, N95 masks, work caps, and goggles/protective screens) to avoid cross infection.
- Patients’ temperature should be checked first, and only those with a normal temperature could enter the waiting area. Patients in the waiting area should be seated more than 1 m apart. During the consultation, the number calling system should be used with strict implementation of “one doctor, one patient, and one consultation room” with no attendants allowed. Patients and their family members should wear surgical masks and keep a distance.
- The medical staff doing ECG or other noninvasive imaging is in close contact with patients and thus should have proper protection.
- All patients should undergo COVID-19 screening immediately (nasal/pharyngeal swabs, CBC, CRP, urea, creatinine, SGOT, SGPT, PT, PTT, INR, cardiac troponin, CXR, CT chest) [12, 13].

### ER management of cardiac patient

**Fig. 1:**
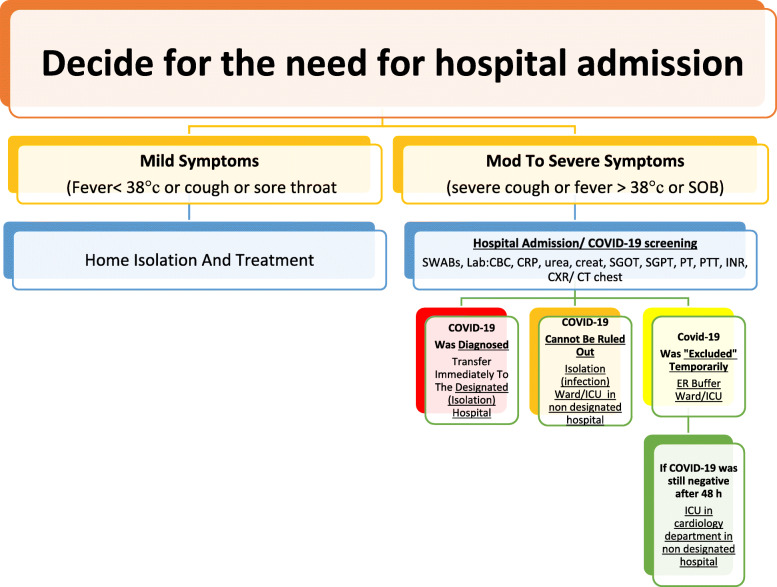
Management of cardiac patient with respiratory symptoms (suspected COVID-19) in a non-designated hospital

- All patients should undergo COVID-19 screening immediately (nasal/pharyngeal swabs, CBC, CRP, urea, creatinine, SGOT, SGPT, PT, PTT, INR, cardiac troponin, CXR, CT chest).
- Decide for the need for hospital admission. No or mild symptoms, home isolation (for 14 days) and no need for hospital admission. Moderate to severe symptoms, hospital admission and isolation (Fig. 1).

A. If COVID-19 was already *diagnosed positive*, he/she would be immediately transferred to the *designated hospital* and followed there by a cardiologist if his cardiac condition is stable. If an urgent cardiac procedure is needed, then it should be done under strict precautions against infection (see management of ACS below).
B. If COVID-19 *cannot be ruled out*, he/she would be transferred to an *isolation ward* in the infectious department for treatment.
C. If COVID-19 was “excluded” temporarily, he/she would be transferred to the *emergency buffer ward* for treatment. Cardiologists will be on duty in the emergency department, the isolation ward and the emergency buffer ward. After admission, these patients would be re-examined for COVID-19 to comprehensively assess whether there was a risk of COVID-19.
D. If COVID-19 was still “excluded,” he/she would be transferred to CCU in the *Cardiology Department.* The CCU ward should adopt the strict principle of single room admission.
E. After 5-7 days of observation in the CCU, a comprehensive assessment of COVID-19 should be performed. If COVID-19 is excluded and the cardiovascular condition is stable, he/she could be transferred to the *regular medicine floor ward* with shared rooms (Fig. 1).

### Cath lab management of cardiac patient

**Fig. 2:**
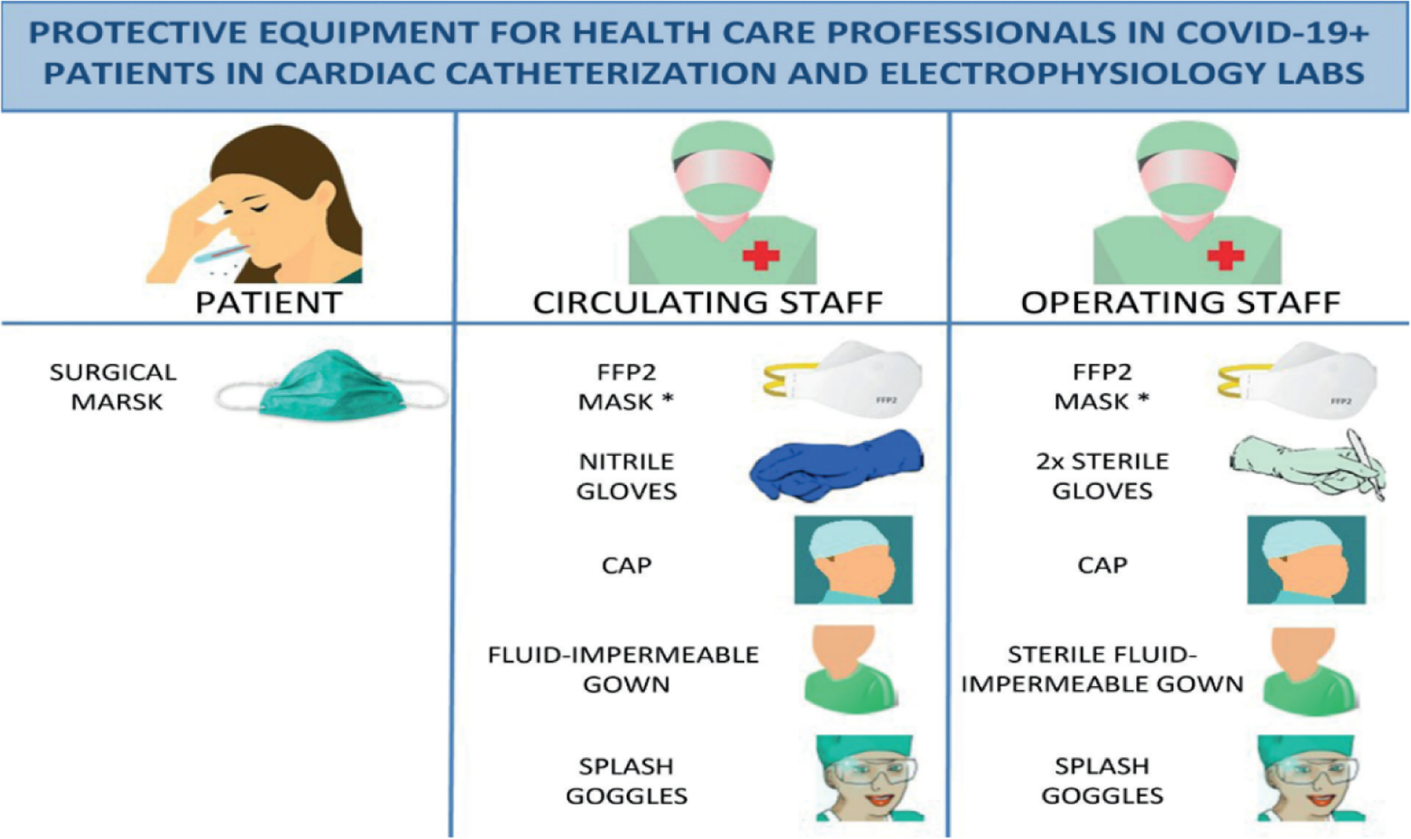
Protective equipment for healthcare professionals in COVID-19+ patients in cardiac catheterization labs. Asterisk denotes for the implantation of pacemakers, ICDs, and transcatheter prostheses, place a surgical mask over the FFP2 mask. FFP2, filtering face piece type 2 [15]

**Fig. 3:**
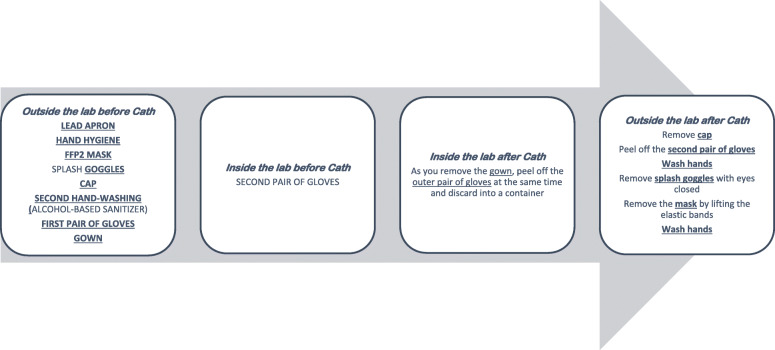
Steps for donning and doffing personal protective equipment for CCL staff

- *Before Entry to Cath Lab*. Minimize pre- and post-procedure waiting times in waiting areas. Use surgical masks in all patients while they wait. Questioning of all patients about respiratory symptoms, fever, and close contacts before entry to the lab; we also recommend temperature-taking in all patients.
- *Allow only essential staff to enter the lab*. Keep doors shut at all times. Patients should put a surgical mask. Prepare drugs before patient entry to the lab. CCLs should prepare COVID-19 carts with all potential supplies for other invasive procedures such as intra-aortic balloon pump, pericardiocentesis, ECMO, and temporary venous pacemakers. Avoid leaving the lab with contaminated equipment (e.g., gown, gloves, and mask) to collect material (e.g., stents and catheters).
- *PPE for operators*. Hand-washing, coated fluid-impermeable gown with cuff (if the gown is not fluid-impermeable, a plastic apron should be added), 2 pairs of gloves, splash goggles or conventional goggles and face shield, cap, and high filtration efficiency FFP2 mask if available (N95/99/100 respirators), (for procedures such as placement of implantable cardioverter-defibrillators, pacemakers, and transcatheter prostheses, a surgical mask should be placed over the FFP2 mask). Closed work shoes are recommended or, if unavailable, boots (or long shoe covers). PPE for staff responsible for transferring patients must wear a fluid-impermeable gown, cap, cuff-covering gloves, goggles, and FFP2 mask (if available) (Figs. 2 and 3).
- Highly infectious patients are those with respiratory symptoms, with confirmed contacts, who may require transesophageal echocardiography, manual ventilation, intubation, or any other type of airway manipulation, unstable patients, especially those with STEMI and during CPR*.*
- High-flow nasal cannula, noninvasive positive pressure ventilation, and use of an Ambu bag should be avoided. If oxygen is required, a mask should be placed over the nasal cannula or oxygen mask.
- Performing endotracheal intubation in the CCL should be avoided; better prior to transfer to the CCL in order to minimize aerosolization. All personnel not essential to intubation should exit the room.
- *After the procedure*. Discard all material used in the procedure in a group III container for biomedical waste and then seal the container. Patients must wear a surgical mask during transfer to the ward or referral center and the orderly or physician (if required) must wear an FFP2 mask.
- Meticulous deep cleansing and disinfection after catheterization procedures are important components of infection control. We recommend using sodium hypochlorite at a concentration of 1000 parts per million, leaving it in contact with the surface for 5 min. UV light-based disinfection may also be a reasonable strategy to employ. Cleaners should be equipped with personal protection equipment. Labs should be cleaned at least 1 h after the procedure, rather than immediately, to allow aerosol deposition. Thorough cleaning procedures may require extra time; therefore, if feasible, such cases should be performed as the final procedure of the day [14–16].

### Management of emergent cardiovascular diseases during COVID-19 pandemic

**Fig. 4:**
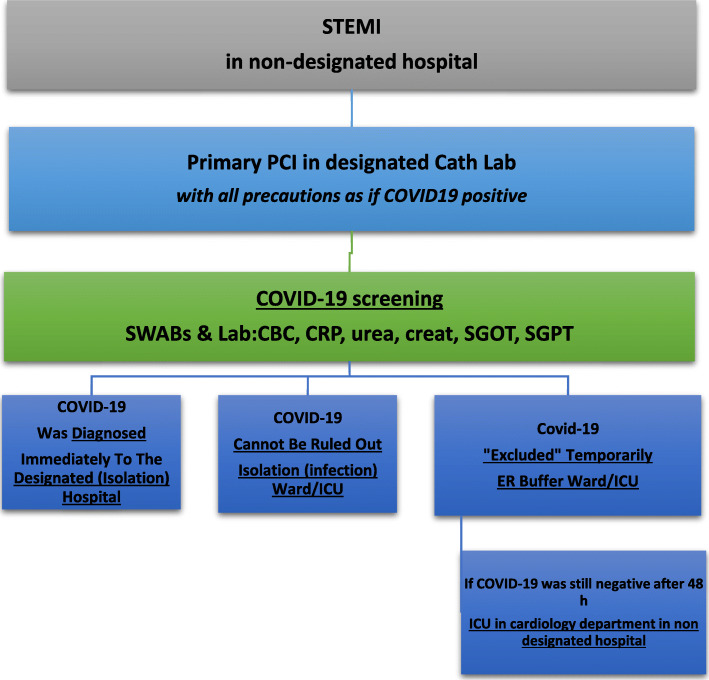
STEMI management if Primary PCI is the standard strategy

**Fig. 5:**
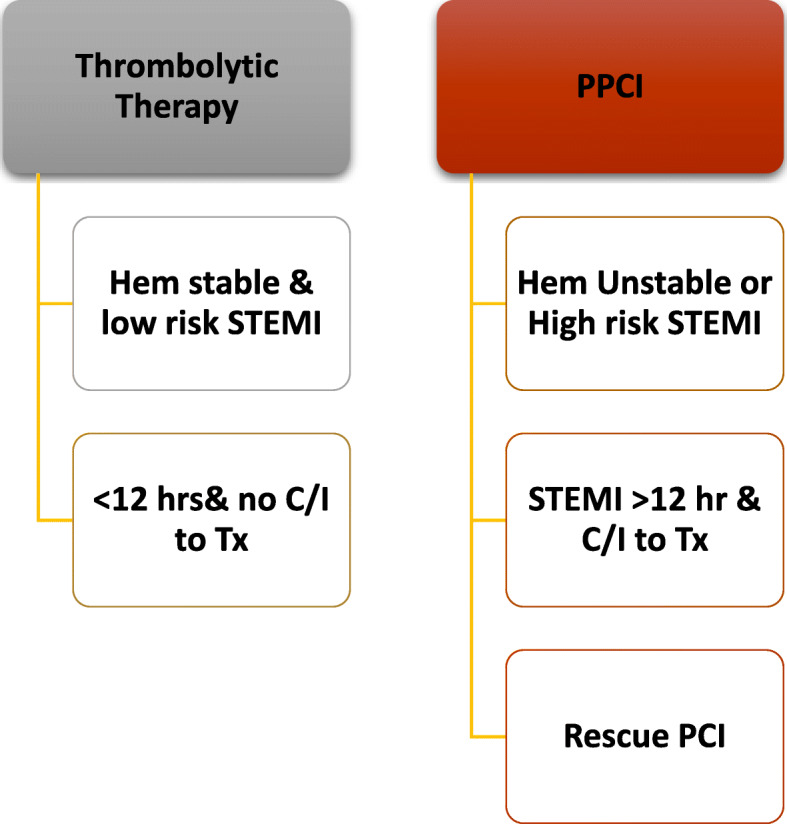
STEMI management if Thrombolytic therapy is the standard strategy After reperfusion (whether by PPCI or lytic or conservative), patients will be transferred to isolation if COVID-19 still under investigation. Only those with twice negative PCR swabs will be transferred to cardiology CCU or ward. Patients with positive COVID-19 are immediately transferred to COVID-19 designated hospitals

**Fig. 6:**
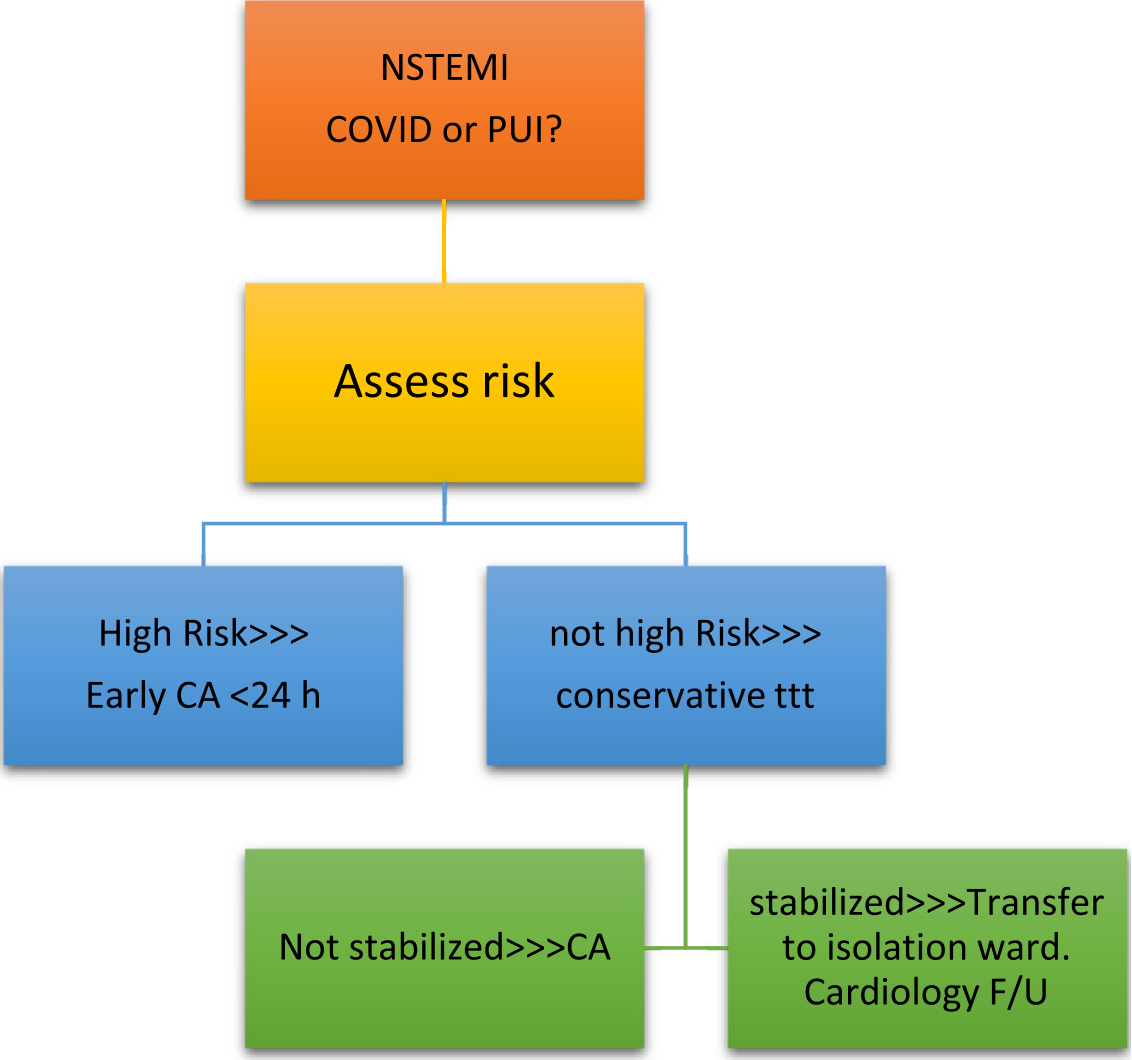
NSTEMI management according to risk stratification

- Some experts suggest that if the hospital is still not overwhelmed, all STEMI patients should be brought for primary PCI. However, if the prevalence of COVID-19 increases causing overburden of the health system resources, the primary PCI options may have to change to be thrombolytic therapy. PCI should only be performed to the culprit vessel unless a non-culprit lesion is deemed unstable or multiple culprit lesions are present (Fig. 4).
- Other experts recommend fibrinolytic therapy (except for anterior STEMI or cardiogenic shock), a potential downside is that these patients then often require prolonged ICU level of care and may end up utilizing vital finite resources. Primary PCI is reserved for high-risk STEMI patients, e.g., those with anterior MI (Fig. 5).
- Most experts agree that NSTEMI patients should be triaged according to their risk stratification (Fig. 6).
- Patients with severe emergent cardiovascular diseases for whom hospitalization and conservative medical treatment are recommended during COVID-19 epidemic are mentioned in Table 3, while severe cardiovascular diseases requiring urgent or emergent intervention or surgery are mentioned in Table 4.
- For patients with valvular heart disease, elective valve interventions should be deferred. Patients with acute heart failure should be admitted for medical treatment if considered at very high risk. TAVI can be considered as an alternative to surgical valve replacement.
- Hospital admission is only recommended for bradyarrhythmia complicated with syncope or unstable hemodynamics mandating implantation of a temporary (bedside implantation as far as possible), or, if indicated, permanent pacemaker.
- Urgent ICD implantation is recommended only for secondary prevention following cardiac arrest or syncopal ventricular tachycardia. Primary prevention implants should be risk assessed.
- All devices should have remote monitoring [14–16].

**Table 3:** Patients with severe emergent cardiovascular diseases for whom hospitalization and conservative medical treatment are recommended during COVID-19 epidemic

1. STEMI for whom thrombolytic therapy is indicated.	
2. STEMI patients presenting after exceeding the optimal window of time for revascularization but yet with worsen symptoms, continuous ST-segment elevation, or mechanical complications.	
3. High risk NSTE-ACS patients (GRACE score ≥ 140)	
4. Uncomplicated Stanford type B aortic dissection	
5. Acute pulmonary embolism	
6. Acute exacerbation of heart failure	
7. Hypertensive emergency	

Modified from CSC expert consensus [11]

**Table 4:** Severe cardiovascular diseases requiring urgent or emergent intervention or surgery

1. Acute STEMI with hemodynamic instability	
2. Life-threatening NSTEMI indicated for urgent revascularization.	
3. Stanford type A or complex Type B acute aortic dissection.	
4. Bradyarrhythmia complicated with syncope or unstable hemodynamics mandating implantation of a temporary (bedside implantation as far as possible), or, if indicated, permanent pacemaker.	
5. Pulmonary embolism presenting with hemodynamic instability for whom regular intravenous thrombolytic therapy might lead to excessively risk of intracranial bleeding, and trans-catheter low-dose thrombolysis in the pulmonary artery may be required.	

Modified from CSC expert consensus [11]

## Conclusion

Cardiologists should be prepared in the COVID-19 pandemic. The challenge is in providing the best quality of care despite limited resources while keeping all medical staff as safe as possible. Consider deferring elective procedures whenever possible. All medical staff should undergo rigorous training on infection control and the use of high-quality personal protection equipment. A much conservative approach for ACS patients is recommended in which invasive interventions are reserved for high risk hemodynamically unstable patients. Cardiologists should promote telemedicine in the outpatient setting, prioritize outpatient contacts, and avoid nosocomial dissemination of the virus to patients and healthcare providers. According to the Chinese Society of Cardiology, during the epidemic, the most important principles of treatment should be the following: control the pandemic as the first priority, prompt patient risk assessment, preference for conservative medical therapy rather than invasive procedures, and strict measures to limit infection spread within the hospital and to healthcare workers.

## Data Availability

Not applicable

## Abbreviations

COVID-19: Coronavirus disease 2019
CXR: Chest X-ray
LV: Left ventricle
LVEF: Left ventricular ejection fraction
PCR: Polymerized chain reaction
CT: Computed tomography
RAS: Renin-angiotensin system
PT: Prothrombin time
PTT: Partial thromboplastin time
INR: International normalized ratio
SGOT: Serum glutamic oxaloacetic transaminase
SGPT: Serum glutamic pyruvic transaminase
ACS: Acute coronary syndrome
CCU: Coronary care unit
Cath Lab: Catheterization laboratory
ECMO: Extracorporeal membrane oxygenation
FFP2: Filtering face piece type 2
N95/99/100: Not resistant to oil
STEMI: ST-elevation myocardial infarction
CCL: Cardiac catheterization laboratory
PCI: Percutaneous coronary intervention
*ICU*: *Intensive care unit*
MI: Myocardial infarction
NSTEMI: Non-ST segment elevation
TAVI: Transcatheter aortic valve implantation
ICD: Implantable cardioverter device
CSC: Chinese Society of Cardiology
