# Correction to: Rapid guide to the management of cardiac patients during the COVID-19 pandemic in Egypt: “a position statement of the Egyptian Society of Cardiology”

**DOI:** 10.1186/s43044-020-00073-1

**Published:** 2020-07-06

**Authors:** Sameh Shaheen, Omar Awwad, Khalid Shokry, Magdy Abdel-Hamid, Adel El-Etriby, Hosam Hasan-Ali, Islam Shawky, Ahmad Magdy, Gamila Nasr, Hamza Kabil, Amr Elhadidy, Mohamad Zaki, Ahmad Hegab

**Affiliations:** 1 Faculty Of Medicine, Ain Shams University, Cairo, Egypt; 2 Faculty Of Medicine, Military Academy, Cairo, Egypt; 3 Faculty Of Medicine, Cairo University, Cairo, Egypt; 4 Faculty Of Medicine, Assuit University, Assuit, Egypt; 5 Faculty Of Medicine, Azhar University, Cairo, Egypt; 6 National Heart Institute, Giza, Egypt; 7 Faculty Of Medicine, Suez Canal University, Ismailia, Egypt; 8 Faculty Of Medicine, Banha University, Banha, Egypt

**Correction to: Egypt Heart J (2020) 72:30**

**https://doi.org/10.1186/s43044-020-00061-5**

Following publication of the original article [1], the authors identified an error in the author name of Hosam Hasan-Ali.

The incorrect author name is: Hsam Hasan-Ali

The correct author name is: Hosam Hasan-Ali

The author group has been updated above and the original article [1] has been corrected.
